# Association of Vitamin B12 Levels with Sleep Quality, Insomnia, and Sleepiness in Adult Primary Healthcare Users in Greece

**DOI:** 10.3390/healthcare11233026

**Published:** 2023-11-23

**Authors:** Izolde Bouloukaki, Maria Lampou, Konstantina Maria Raouzaiou, Eirini Lambraki, Sophia Schiza, Ioanna Tsiligianni

**Affiliations:** 1 Department of Social Medicine, School of Medicine, University of Crete, 71410 Heraklion, Greece; med3933@edu.med.uoc.gr (M.L.); med3975@edu.med.uoc.gr (K.M.R.); lampraki@msn.com (E.L.); i.tsiligianni@uoc.gr (I.T.); 2 Sleep Disorders Center, Department of Respiratory Medicine, School of Medicine, University of Crete, 71410 Heraklion, Greece; schizas@uoc.gr

**Keywords:** sleep disorders, primary care, vitamin B12, insomnia symptoms, excessive daytime sleepiness, sleep quality

## Abstract

Despite vitamin B12’s recognized importance for the nervous system, there is still a lack of research on the association between vitamin B12 and sleep, especially in primary care settings. We assessed vitamin B12 levels in adult primary healthcare users and investigated correlations with sleep quality, insomnia, and sleepiness. In this cross-sectional study, 512 consecutive participants were included. Information regarding anthropometrics, socio-demographics, and medical history was obtained. The Epworth Sleepiness Scale (ESS), Athens Insomnia Scale (AIS), and Pittsburg Sleep Quality Index (PSQI) were used to quantify excessive daytime sleepiness (EDS), insomnia symptoms, and sleep quality, respectively. The median vitamin B12 was 342 (266, 446) pg/mL. After adjustments, vitamin B12 levels < 342 pg/mL showed significant associations with insomnia symptoms [OR (95% CI) 2.434 (1.331–4.452), *p* = 0.004], especially in elderly, non-obese, and female participants, with EDS only in obese participants [OR (95% CI) 3.996, (1.006–15.876), *p* = 0.039]. Nonetheless, there was no significant association between B12 levels and poor sleep quality (OR 1.416, 95% CI 0.678–2.958, *p* = 0.354). In conclusion, our results show that lower vitamin B12 was associated with insomnia symptoms and sleepiness in specific groups of participants. However, further research with objective measurements of sleep is crucial to assess the relationship between sleep and vitamin B12.

## 1. Introduction

Sleep health involves a complex sleep and wakefulness pattern that is tailored to individual, social, and environmental demands, with the goal of enhancing physical and mental well-being [1]. However, prior research has indicated that a significant proportion of the general population experiences sleep impairment, including insufficient sleep duration, prolonged sleep latency, frequent and prolonged nocturnal awakenings, and other sleep disruptions [2,3,4,5,6]. Specifically, it appears that approximately one-third of adults in primary care settings report symptoms of insomnia [7]. Moreover, among adults aged 65–79, more than a quarter get less than the recommended 7 h of sleep, while over 40% experience poor sleep quality, 13% have frequent insomnia, and 9% suffer from daytime fatigue and symptoms suggestive of sleep apnea [8]. Sleep impairment over time has been associated with a variety of health conditions, including metabolic and cardiovascular diseases, cancer, depression, as well as greater risk of mortality [9,10,11,12,13]. In light of the unfavorable outcomes of sleep impairment, it is crucial to identify and explore the potential associated modifiable factors.

Recently, there has been a growing interest in the importance of diet and nutrition in relation to sleep impairment. Evidence suggests that there is a reverse connection between dietary or serum macro- and micronutrient levels and sleep disturbances [14,15,16]. More specifically, macronutrients appear to play a role in modulating neurotransmitter levels and impacting intrinsic sleep mechanisms, ultimately influencing sleep patterns [17,18]. Moreover, micronutrients may impact the activity of presynaptic neurons or the synthesis of sleep-regulating neurotransmitters, including serotonin, N-methyl-d-aspartate (NDMA) glutamate, and melatonin [18,19,20]. However, the clinical relevance of micronutrients to sleep health has received less attention, and findings regarding some micronutrients, such as vitamin B12, remain inconsistent [21].

Vitamin B12, also known as cobalamin, is a vital vitamin for the nervous system that cannot be synthesized by the body and must be obtained through dietary consumption from animal products like liver, fish, eggs, or dairy products [22]. It seems to play a crucial role in melatonin synthesis which regulates sleep rhythms with the potential to improve the sleep–wake cycle [20]. Therefore, it is a micronutrient worth considering when examining modifiable factors that are linked to sleep disturbances. However, there are few studies exploring the relationship between serum vitamin B12 and sleep health [21,23,24]. Despite the lack of conclusive evidence in earlier studies [20,25,26,27], more recent research points toward a negative correlation between serum vitamin B12 levels and sleep duration [23,28]. Moreover, most of the research has predominantly focused on the correlation between B12 and the duration of sleep, and, overall, there is a scarcity of information regarding the impact of B12 on the quality of sleep, symptoms of insomnia, and sleepiness.

The aforementioned findings require more investigation, especially for populations without pertinent data, like Greece. Therefore, we hypothesized that vitamin B12 levels are associated with sleep quality, insomnia symptoms, and daytime sleepiness. To test this hypothesis, we assessed serum vitamin B12 levels in adult primary healthcare users in Crete, Greece, and investigated possible correlations with socio-demographic factors, co-morbidities, sleep quality, insomnia symptoms, and daytime sleepiness, after considering other confounders.

## 2. Materials and Methods

### 2.1. Patients

In this single-center, cross-sectional study, patients aged ≥18 years were consecutively approached by General Practitioners (GPs) during their regular consultations at four public primary healthcare practices (one organized health center and three satellite practices) located in rural and semi-urban areas, in the region of Crete over a 3-year period (2019–2022). The exclusion criteria included a history of current infectious diseases, gastrointestinal reabsorption disorders, bariatric surgery, pregnant or breastfeeding women, diagnosed sleep disorders or on sleep medication, intake of vitamin B12 supplements or medications with known effects on serum vitamin B12 levels, or adherence to vegan diet. Ethical approval was provided by the Health Regional Administration (DYPE) of Crete Scientific Board Ethics Committee (protocol number 23542/06-12-2017) and the patients gave written informed consent.

### 2.2. Demographic Characteristics

The collected data included anthropometric parameters, such as age, gender, height, weight, body mass index (BMI), details of co-morbidities, smoking history, and alcohol intake. We determined the patient’s height with a decimal point precision of 0.1 cm. The body weight was measured using an electronic scale that had a maximum capacity of 200 kg. The participant’s BMI was determined by dividing their weight in kilograms by the square of their height in meters (kg/m^2^), and obesity was classified as a binary variable using standard BMI criteria (non-obese: BMI  <  30, obese: BMI  ≥  30) [29]. The Epworth Sleepiness Scale (ESS) was utilized to assess subjective excessive daytime sleepiness (EDS) [30]. The scale measures from 0 to 24 and a value less than or equal to 10 is considered normal. The evaluation of insomnia was performed utilizing the Athens Insomnia Scale (AIS), an 8-item self-assessment psychometric instrument that has been employed as a means to assess the severity of insomnia [31]. The range of total scores falls between 0 and 24, and insomnia is indicated by a total score of 6 or above. The Pittsburgh Sleep Quality Index (PSQI), a 19-item self-rated questionnaire, was employed to assess sleep quality. It evaluates subjective sleep quality and quantity, sleep habits that impact quality, and sleep disturbance occurrence in adults over a 1-month period. A global score of 6 or higher indicates poor sleep (range 0–21) [32].

### 2.3. Vitamin B12 Measurement

Serum samples for vitamin B12 levels were obtained. Blood samples were taken after at least 8 hr of fasting, after which they were immediately centrifuged (3000 rpm for 10 min), and the serum that was obtained was frozen at −80 °C until processing. Serum vitamin B12 levels were measured using the Alinity i system (Abbott Laboratories, Chicago, IL, USA) chemiluminescent microparticle immunoassay (CMIA) analyzer. For this study, the data on serum vitamin B12 levels were divided into two groups based on the median value of its measurement.

### 2.4. Statistical Analysis

The results are presented as the mean ± standard deviation (SD) for continuous variables if normally distributed, and as the median (25th–75th percentile) if not. Qualitative variables are presented as absolute number (percentage). For comparisons between groups, a two-tailed t-test for independent samples (for normally distributed data) or a Mann–Whitney U-test (for non-normally distributed data) was utilized for continuous variables and the Chi-square test for categorical variables. Logistic regression analysis was applied to examine the effect of vitamin B12 levels on co-morbidities, sleepiness, sleep quality, and insomnia symptoms, after controlling for potential explanatory variables, including age, gender, BMI, smoking status, co-morbidities, marital, educational status, menopausal status, and alcohol intake. We checked multicollinearity among the predictors using collinearity statistics to ensure that collinearity between predictor variables was in the acceptable range as indicated by the tolerance value variance inflation factor. For the purpose of this analysis, the term cardiovascular disease, used as a predictor in logistic regression models, referred to any of the following conditions: coronary disease, atrial fibrillation cerebrovascular disease, and heart failure. Age was considered continuously and categorically, as age groups of 18–59 and >60 years, BMI was also considered continuously and categorically, as BMI groups of <30 and ≥30 kg/m^2^. The results were considered significant when *p*-values were <0.05. Data were analyzed using SPSS software (version 25, SPSS Inc., Chicago, IL, USA).

## 3. Results

### 3.1. Study Population

**Figure 1:**
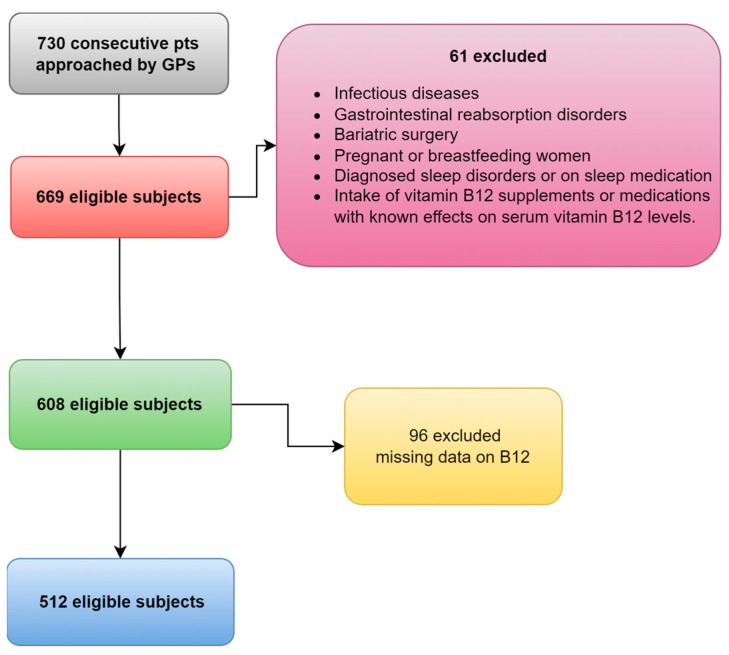
The flowchart of patients finally included.

Following the screening process, a grand total of 512 participants (whose average age was 64 years and of which 35% were male) were deemed eligible and subsequently included in the study, as depicted in Figure 1. Among the participants, 27% reported excessive daytime sleepiness (ESS ≥ 11), while 40% experienced insomnia symptoms (AIS ≥ 6) and 37% had poor sleep quality (PSQI ≥ 6). The median level of vitamin B12 was 342 (266, 446) pg/mL and there was no statistically significant difference observed between genders (349 vs. 326, *p* = 0.108). Participants were divided into two groups based on the median value of vitamin B12: those with B12 levels below (<) 342 pg/mL, recognized as low levels, and those with (≥) 342 pg/mL levels, recognized as normal levels.

Table 1 displays the baseline characteristics of the study population, stratified by vitamin B12 status. Patients with low vitamin B12 levels did not exhibit any notable differences in baseline characteristics compared to those without low levels, including age, gender, menopausal status, BMI, comorbidities, and smoking status (all *p* > 0.05).

### 3.2. Vitamin B12 Correlations with Sleep Quality, Daytime Sleepiness, and Insomnia Symptoms

**Figure 2:**
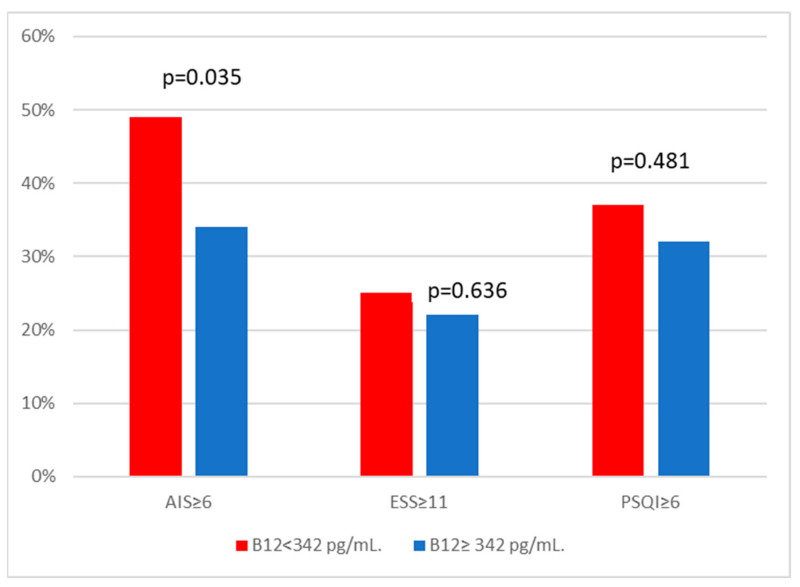
Comparison of questionnaires scores based on vitamin B12 levels. AIS: Athens Insomnia Scale; ESS: Epworth Sleepiness Scale; PSQI: Pittsburgh Sleep Quality Index.

Table 2 and Figure 2 demonstrate differences in questionnaire scores between people with low vitamin B12 levels and those without. The groups showed no significant variation in ESS score (6 vs. 6, *p* = 0.908) or excessive daytime sleepiness (ESS > 10) (25 vs. 22%, *p* = 0.636). The PSQI score failed also to exhibit any significant differences between the groups (5 vs. 4, *p* = 0.286). Additionally, the difference in the percentage of people with PSQI ≥ 6 between the groups was not statistically significant (37 vs. 32, *p* = 0.286).

On the other hand, individuals with low levels of vitamin B12 exhibited a greater AIS score, albeit statistically insignificant, compared to those who did not (5 vs. 4, *p* = 0.419). However, there was a significant difference between the two groups regarding the percentage of people with AIS ≥ 6 (49 vs. 34%, *p* = 0.035). In addition, the study revealed that participants who experienced insomnia symptoms had a notably lower level of vitamin B12 compared to those who did not report such symptoms (310 vs. 354, *p* = 0.03).

Table 3, Table 4 and Table 5 show multiple stepwise logistic regression analysis of the relationship between excessive daytime sleepiness (EDS), sleep quality, and insomnia symptoms and various independent variables, after multiparametric adjustments for age, gender, obesity, co-morbidities and marital, educational, menopausal and smoking status. The prevalence of EDS was significantly predicted by obesity and male gender in the population studied (Table 3). Although low vitamin B12 levels did not have a significant predictive value for EDS, there was a significant correlation between low vitamin B12 levels and EDS in obese patients (BMI ≥ 30) (OR = 3.996, 95% CI = 1.006–15.876, *p* = 0.039).

The occurrence of insomnia symptoms was found to be linked with female gender, hypertension, and insufficient vitamin B12 levels (Table 4). Further analysis of the subgroups that were stratified by gender, age, and BMI indicated that low vitamin B12 levels were significantly associated with insomnia symptoms only in the subgroup of elderly (age group > 60 years) (OR = 3.197, 95% CI = 1.495–6.836, *p* = 0.003), females (OR = 2.549, 95% CI = 1.257–5.169, *p* = 0.009), and non-obese subjects (BMI group < 30) (OR = 2.955, 95% CI = 1.386–6.300, *p* = 0.005).

There was no correlation found between vitamin B12 levels and poor sleep quality (Table 5), even after controlling for confounding factors. Furthermore, subgroup analyses based on gender, age, and BMI failed to reveal any significant associations.

It is worth noting that only 42 (8.2%) and 7 (1.3%) participants demonstrated high (>600 pg/mL) and very high (>900 pg/mL) levels of serum vitamin B12. None of these participants had previously been diagnosed with cancer, liver, or kidney disease. While the number is small, making comparisons less reliable, these participants did not display a greater prevalence of sleep disorders than other participants with lower vitamin B12 levels.

## 4. Discussion

Our study aimed to investigate the potential correlation between vitamin B12 levels and subjective sleep symptoms as assessed by ESS, AIS, and PSQI questionnaires. We found that low vitamin B12 levels were associated with a 2.4-times risk increase of insomnia symptoms and the association was significant in the older, female, and non-obese subjects. Furthermore, in obese subjects, a significant association of excessive daytime sleepiness and low vitamin B12 levels was noted.

Despite the acknowledged significance of vitamin B12 for the nervous system and the risks of its deficiency, there remains an insufficiency of research on the relationship between vitamin B12 and sleep disorders, including the specific blood level thresholds that could trigger such disorders. The existing studies which have focused primarily on the relationship between micronutrients and sleep duration have yielded inconsistent results regarding the impact of vitamin B12 on sleep patterns [21]. Initially, studies did not report any significant or conclusive impact of vitamin B12 on the duration and phase of nocturnal sleep [20,25,26], except for Mayer et al. [27], who observed a stimulating effect of vitamin B12 supplementation associated with decreased sleep. Subsequent research indicated that there is a potential negative correlation between serum vitamin B12 concentrations and the duration of sleep [23,28]. It is also worth noting that elevated levels of vitamin B12 should be taken into account during the diagnostic process, as they may indicate an underlying medical condition such as liver or kidney disease or cancer [33]. High levels of vitamin B12 may also be paradoxically linked to functional deficiency due to impairments in the uptake and function of vitamin B12 within tissues [34]. Nonetheless, in our study, only a minority of patients displayed high levels of vitamin B12 and did not exhibit a greater prevalence of sleep disorders than other participants with lower vitamin B12 levels.

The available evidence regarding the correlation of levels of vitamin B12 and the likelihood of experiencing symptoms of insomnia is even more limited. Using data from 2459 adults and conducting cross-sectional surveys of the health and nutritional status of the American population, the National Health and Nutrition Examination Surveys (NHANES) study concluded that vitamin B12 levels and the duration of sleep are inversely associated [23]. Nevertheless, no association was observed between vitamin B12 and symptoms of insomnia in this population; however, one must bear in mind that insomnia was assessed using only one question requiring a yes or no response. Similarly, in a subsequent study that examined 575 patients over the age of 65 from a geriatric population, no significant difference was found between levels of serum vitamin B12 and the severity of insomnia assessed by the Insomnia Severity Index (ISI) [35]. In our study, a 2.4-fold increase in the odds ratio for insomnia symptoms was observed in individuals with low vitamin B12 levels, demonstrating a significant association between the two. These findings appear to align with a previous investigation involving 355 Arabian female students, which discovered a negative association between serum vitamin B12 levels and sleep latency. Participants in the same study exhibiting higher serum vitamin B12 levels ranging from 333.1 to 482.2 also reported a decreased use of sleep medication [24]. In addition, a lower intake of vitamin B12 seemed to be associated with a delayed sleep–wake rhythm [28] and a higher prevalence of insomnia symptoms, assessed by the Insomnia Screening Questionnaire [36]. The crucial function of B12 in sleep latency is reinforced by a previous study that examined the impact of vitamin B12 supplementation on the sleep–wake cycle of individuals with delayed sleep phase syndrome, showing a significant temporary improvement in the supplemented group [26]. The therapeutic benefits of vitamin B12 supplementation in sleep–wake disorder management were also suggested by Maeda et al., who noted its role in regulating circadian rhythms [37]. Notably, the link between insufficient vitamin B12 levels and symptoms of insomnia was more prominent in the subgroup of non-obese subjects. Vitamin B12 might affect sleep in a distinct way for non-obese adults. Nevertheless, the constraints of the cross-sectional study design and the lack of relevant data from literature prohibit any speculation on the potential reasons for these variations in observations.

On the other hand, our findings contradict those of a recent study involving 418 Chinese participants with type 2 diabetes, which identified an independent positive association between vitamin B12 levels and insomnia symptoms, as assessed by AIS, after controlling for confounding variables [38]. Our speculation is that this discrepancy may have arisen partly due to variations in race/ethnicity. A recent investigation supports this assertion, revealing that a combination of genetic and acquired/environmental factors contribute to the ethnic differences in serum vitamin B12 levels [39]. Additionally, Chinese participants demonstrate a higher probability of suffering from insufficient sleep (<6 h) and lower occurrences of insomnia symptoms and daytime sleepiness when compared to white patients [40].

Interestingly, the association of B12 and insomnia symptoms was more pronounced in elderly, females, and non-obese participants. Despite the lack of explanation currently, it is plausible that vitamin B12 affects sleep in distinct ways for both older and younger adults, due to the notable changes in sleep structure with aging. Variances in circadian phase, light responsiveness, and clock gene expression identified between older and younger adults [41], could lead to a different reaction to vitamin B12.

Our investigation also revealed that vitamin B12 levels did not correlate with sleep quality as measured by PSQI in our cohort. These results are in agreement with prior investigations conducted on Chinese individuals with type 2 diabetes [38], and female Arab students [22], wherein no correlation was found between serum vitamin B12 levels and sleep quality, as assessed by PSQI. The NHANES study also did not identify a link between vitamin B12 levels and metrics of sleep quality [23]. Additionally, 14 healthy adult participants did not exhibit any association between their serum vitamin B12 levels and actigraphy-assessed sleep parameters before and two weeks after receiving 3 mg/day cyanocobalamin supplementation [42]. Furthermore, research that has focused on the dietary intake of vitamin B12 and its effect on sleep quality has yielded conflicting findings [43,44]. A correlation was noted by Condo et al. between vitamin B12 consumption and improved sleep quality, evaluated by actigraphy in 32 female athletes at the elite level [43]. Conversely, Jahrami et al. found a conflicting outcome, indicating a strong link between daily vitamin B12 intake and lower sleep quality, evaluated by PSQI in 96 healthy individuals [44]. Given that these studies utilized methods to assess dietary intake as a means of measuring vitamin B12 status, this may have resulted in an unreliable memory of individual dietary habits or errors in measurement, which could have influenced the final measurements of the aforementioned connections. As a result, more studies are warranted to explore the potential benefits of high serum levels or intake of vitamin B12 on sleep quality.

To date, few data exist regarding vitamin B12 status in the adult population and prevalence of EDS. According to our study, low levels of vitamin B12 were not found to significantly predict EDS, which is consistent with prior studies including the NHANES study (n = 2459) [23] and also with studies that focused on elderly patients with chronic kidney disease (n = 367) [45] and elderly patients with or without dementia (n = 800) [46]. However, we found a significant correlation between low vitamin B12 levels and EDS in obese participants. The exploration of a plausible association between low vitamin B12 levels as a cause of EDS in obese population is constrained. Within the literature, a singular case is reported of an obese patient with obstructive sleep apnea (OSA) who experienced excessive daytime sleepiness (EDS) despite receiving optimal treatment and having a severe deficiency in vitamin B12. The intake of vitamin B12 supplements in this case proved to reverse EDS [47], which could imply that vitamin B12 deficiency may play a role in causing daytime sleepiness.

The results from the current study have important implications for primary care health practice. Our study’s overall sample revealed that a significant number of participants reported insomnia symptoms, poor sleep quality, and EDS, indicating that primary care physicians encounter challenges in identifying and managing these disorders. Vitamin B12 levels < 342 were identified as a significant predictor of a high AIS score, particularly in individuals over 60, females, and those who are not obese, and also a significant predictor of high ESS score in obese subjects. Therefore, particularly within this subset of individuals, these vitamin B12 levels may aid in distinguishing adults with a higher likelihood of developing insomnia and sleepiness later on.

It is plausible that several limitations might have affected our results. First, the cross-sectional design of our study precludes us from assigning causality to the associations between insomnia symptoms, excessive daytime sleepiness, and vitamin B12. Second, self-reported sleep measures were employed to determine sleep disorders, which may be affected by estimation bias, compared to more-objective measures such as actigraphy and polysomnography. Thirdly, we had to exclude patients with specific characteristics based on justified exclusion criteria to minimize potential bias in the study’s findings. While we accounted for a significant number of relevant confounding variables, there is still the possibility of unmeasured confounders that might impact our results, such as dietary habits, including food history and stress levels. Nevertheless, no noteworthy dissimilarities are expected concerning dietary patterns, given that our research comprised of only Cretan participants, dwelling in the same area, with similar dietary habits. Furthermore, the use of BMI fails to distinguish between fat mass and lean mass, thus the analysis conducted does not include body composition analysis [48]. Lastly, given that the participants were residents of Crete (located in the southern region of Greece), the generalization of our conclusions to all Greek primary care users should be made with caution.

## 5. Conclusions

Our results showed that lower vitamin B12 levels were associated with a higher risk of insomnia symptoms and EDS in specific subgroup of adult primary healthcare users. Despite the need for additional work to elucidate the potential underlying mechanisms of these associations, low vitamin B12 levels should be considered as a possible culprit in certain subpopulations of patients experiencing sleep disturbances. Further prospective studies are also needed to determine the accurate levels of vitamin B12 for sleep health and the need for vitamin B12 supplementation.

## Figures and Tables

**Table 1:** Clinical characteristics of the 512 primary care users according to vitamin B12 status.

		Total Population According to Vitamin B12 Status		
	Total Population	Vitamin B12 ≥ 342 pg/mL	Vitamin B12 < 342 pg/mL	*p*-Value
	N = 512	N = 256	N = 256	
**Demographics**				
Gender, males (%)	180 (35%)	84 (33%)	96 (38%)	0.343
Menopause (%)	249 (75%)	129 (75%)	120 (75%)	0.997
Age, years	64 ± 16	63 ± 16	65 ± 15	0.144
Age ≥ 60 years	323 (63%)	151 (59%)	172 (67%)	0.160
BMI (kg/m^2^)	30 ± 16	30 ± 19	30 ± 11	0.903
BMI ≥ 30, n (%)	168 (33%)	84 (33%)	84 (33%)	0.966
Smoking status				
Never, n (%)	272 (53%)	148 (58%)	124 (49%)	
Current/, n (%)	107 (21%)	56 (22%)	51 (20%)	
Former, n (%)	133 (26%)	51 (20%)	82 (32%)	0.066
**Co-morbidities**				
Hypertension	260 (51%)	130 (51%)	130 (51%)	0.999
Diabetes Type 2	97(19%)	38 (15%)	59 (23%)	0.128
Hyperlipidemia	279 (55%)	133 (52%)	146 (57%)	0.463
COPD	38 (7%)	18 (7%)	20 (8%)	0.163
Asthma	20 (4%)	15 (6%)	5 (2%)	0.136
Coronary artery disease	35 (7%)	20 (8%)	15 (6%)	0.529
Atrial fibrillation	23 (5%)	13 (5%)	10 (4%)	0.837
Cerebrovascular disease	11 (2%)	7 (3%)	4 (1%)	0.429
Cardiovascular disease	74 (15%)	43 (17%)	31 (12%)	0.216
Depression (on medication)	59 (11%)	33 (13%)	26 (10%)	0.533

Data are presented as mean values ± SD or median (25th–75th percentile), unless otherwise indicated. BMI, body mass index; COPD, chronic obstructive pulmonary disease; cardiovascular disease: coronary heart disease or atrial fibrillation or cerebrovascular disease or heart failure.

**Table 2:** Questionnaires scores of the 512 primary care users according to vitamin B12 status.

		Total Population According to Vitamin B12 Status		
	Total Population	Vitamin B12 ≥ 342 pg/mL	Vitamin B12 < 342 pg/mL	*p*-Value
	N = 512	N = 256	N = 256	
**Daytime sleepiness**				
ESS	6 (4, 10)	6 (4, 10)	6 (4, 10)	0.908
ESS ≥ 11 (%)	120 (23%)	56 (22%)	64 (25%)	0.636
**Insomnia symptoms**				
Athens Insomnia Scale Score	5 (3, 6)	4 (3, 6)	5 (3, 6)	0.419
Athens Insomnia Scale Score ≥ 6 (%)	212 (41%)	87 (34%)	125 (49%)	0.035
**Sleep Quality**				
PSQI	5 (3, 6)	4 (3, 6)	5 (3, 6)	0.286
PSQI ≥ 6	177 (35%)	82 (32%)	95 (37%)	0.481

Data are presented as mean values ± SD or median (25th–75th percentile), unless otherwise indicated. ESS, Epworth Sleepiness Scale; PSQI, Pittsburgh Sleep Quality Index.

**Table 3:** Multiple stepwise logistic regression analysis of the relationship between excessive daytime sleepiness (ESS ≥ 11) and various independent variables.

	B	S.E.	*p*-Value	OR (95%CI)
**Males versus females**	1.098	0.385	0.004	2.998 (1.410–6.374)
**Age > 60 years**	−0.169	0.366	0.669	0.844 (0.388–1.835)
**Body mass index ≥ 30**	0.759	0.342	0.027	2.135 (1.091–4.178)
**Current/former smoking**	0.362	0.397	0.362	1.436 (0.660–3.126)
**Hypertension**	0.139	0.393	0.723	1.159 (0.532–2.484)
**Diabetes type 2**	0.443	0.429	0.302	1.557 (0.671–3.614)
**Cardiovascular disease**	−0.554	0.521	0.288	0.575 (0.207–1.597)
**COPD**	0.189	0.546	0.729	1.208 (0.414–3.521)
**Depression**	−0.050	0.573	0.931	0.952 (0.310–2.924)
**Vitamin B12 < 342**	0.101	0.517	0.762	1.106 (0.576–2.125)

ESS: Epworth Sleepiness Scale; COPD: chronic obstructive pulmonary disease.

**Table 4:** Multiple stepwise logistic regression analysis of the relationship between insomnia symptoms (AIS ≥ 6) and various independent variables.

	B	S.E.	*p*-Value	OR (95%CI)
**Females versus males**	1.266	0.397	0.001	3.547 (1.630–7.718)
**Age > 60 years**	−0.129	0.375	0.730	0.879 (0.422–1.831)
**Body mass index ≥ 30**	0.073	0.318	0.818	1.076 (0.577–2.009)
**Current/former smoking**	0.227	0.356	0.523	1.255 (0.625–2.520)
**Hypertension**	1.229	0.368	0.001	3.419 (1.661–7.035)
**Diabetes type 2**	−0.467	0.424	0.270	0.627 (0.273–1.438)
**Cardiovascular disease**	0.526	0.453	0.246	1.692 (0.696–4.112)
**COPD**	0.133	0.603	0.826	1.142 (0.350–3.724)
**Depression**	−0.467	0.472	0.322	0.627 (0.249–1.579)
**Vitamin B12 < 342**	0.890	0.308	0.004	2.434 (1.331–4.452)

AIS: Athens Insomnia Scale; COPD: chronic obstructive pulmonary disease.

**Table 5:** Multiple stepwise logistic regression analysis of the relationship between poor sleep quality (PSQI ≥ 6) and various independent variables.

	B	S.E.	*p*-Value	OR (95%CI)
**Females versus males**	0.318	0.460	0.489	1.374 (0.558–3.384)
**Age > 60 years**	0.385	0.469	0.410	1.470 (0.588–3.679)
**Body mass index ≥ 30**	0.305	0.424	0.471	1.357 (0.591–3.116)
**Current/former smoking**	−0.624	0.424	0.141	0.536 (0.233–1.229)
**Hypertension**	0.028	0.438	0.948	1.029 (0.436–2.430)
**Diabetes type 2**	0.275	0.522	0.599	1.316 (0.473–3.662)
**Cardiovascular disease**	0.262	0.592	0.658	1.300 (0.408–4.144)
**COPD**	1.307	0.767	0.089	3.693 (0.821–16.614)
**Depression**	0.540	0.631	0.392	1.716 (0.498–5.918)
**Vitamin B12 < 342**	0348	0.376	0.354	1.416 (0.678–2.958)

PSQI: Pittsburgh Sleep Quality Index; COPD: chronic obstructive pulmonary disease.

## Data Availability

The datasets generated during and/or analyzed during the current study are available from the corresponding author on reasonable request.
